# Ten simple rules for Global North researchers to stop perpetuating helicopter research in the Global South

**DOI:** 10.1371/journal.pcbi.1009277

**Published:** 2021-08-19

**Authors:** Danny Haelewaters, Tina A. Hofmann, Adriana L. Romero-Olivares

**Affiliations:** 1 Department of Biology, Ghent University, Ghent, Belgium; 2 Faculty of Science, University of South Bohemia, České Budějovice, Czech Republic; 3 Herbario UCH, Universidad Autónoma de Chiriquí, David, Panama; 4 Department of Biology, New Mexico State University, Las Cruces, New Mexico, United States of America; Carnegie Mellon University, UNITED STATES

## Introduction

The practice of Global North (i.e., “richer” globalized countries located in the northern hemisphere, except for Australia and New Zealand) researchers making roundtrips to the Global South (i.e., “poorer” developing countries located around the tropics and in the Southern hemisphere) to collect materials and then process, analyze, and publish results with little to no involvement from local collaborators is referred to as “helicopter research” or “parachute research” [1]. At best, local scientists provide logistical help and knowledge of the local community, such as field site guiding, identification of local organisms, translation from and to local languages, and facilitating resources to foreign scientists. However, often, these necessary actors in the scientific process receive little to no retribution for their work and knowledge. For example, a systematic problem in academia is that local scientists and graduate and undergraduate students are often not offered coauthorship in manuscripts for which their contributions were essential (e.g., project planning, logistics, and knowledge of local biodiversity). Even worse is that research remains unavailable for them and others who contributed substantially, since in most cases, peer-review publications are available behind a paywall (but see [2]), and they are written in English, which is the second or third language for many Global South researchers [3]. Furthermore, local communities where Global North scientists come to conduct helicopter research are usually left out of broader impacts and outreach efforts, as these tend to happen in Global North communities.

Helicopter research is a way to perpetuate colonization practices [4], and power imbalances are critical in perpetuating helicopter research. For example, Global North researchers often set the research agenda based on priorities of their funding agencies, which, in many cases, are decided upon by those same researchers sitting in decision-making committees of those very agencies. All too often, proposals are developed without a deep understanding of problems and priorities of Global South countries where the anticipated research will take place. Global South collaborators are rarely invited to brainstorm and set the research agenda for their needs. This opens the door for unequal partnership with research objectives that may be irrelevant for Global South collaborators, who then, are forced to accept an already funded proposal due to lack of research funding within their own countries or institutions [5]. Thus, power and politics behind science are interconnected and are a driving force behind helicopter research.

Helicopter research is a problem that also happens within Global North and within Global South countries, where dominating cultures perpetuate abuse on historically marginalized communities, including those of indigenous people, people of color, people from lower socioeconomic status, etc. This is an increasingly complex problem that has been intensely discussed in other papers and for which extensive guidelines exist [6–14]. Here, we address the problem that exists specifically between researchers from the Global North—often members of the dominating culture—toward people of the Global South who may or may not be members of the dominating culture. We propose 10 simple rules for avoiding helicopter research for better, collaborative, and non-colonial science between the Global North and the Global South.

### Rule 1: Establish “win-win” collaborations and equal partnerships

Collective development of research ideas should be a priority. Often, Global North researchers set the research agenda due to better access to research money compared to researchers in the Global South. Rarely are Global South collaborators involved in the early stages of writing grant proposals (i.e., brainstorming). As a result, they have no say on their research priorities based on local needs. This power imbalance creates unequal partnerships, and it is a driving force of helicopter research. However, Global North and Global South knowledge is most likely complementary; research agendas and projects should be a “win-win,” with both sides benefitting from the collaboration. A division within the Dutch Research Council (NWO), WOTRO Science for Global Development, introduced a 2-stage grant making process that required successful pre-proposals to organize workshops with collaborators and stakeholders in the country where the proposed research would be conducted. Funding for these workshops would be provided and later deducted from the grant if funded. This led to Global South researchers sharing leading roles with Global North researchers and consequently addressing research priorities for both sides of the collaboration and creating closer collaborations once the grant was awarded. Despite some downsides, the advantages outweigh the disadvantages [5]. This is a prime example on how to create “win-win” collaborations and equal partnerships.

Another important aspect of “win-win” collaborations and equal partnerships, especially in academia, is authorship. Collaborative international research is cited the most [15,16], and, presumably, has higher research impact. However, many collaborators in the Global South are left out of publications even though they are integral for field access and data collection. In many cases, their local knowledge is essential for the approval of collecting permits by local authorities and for identification of local biodiversity. Yet, when the time comes to write manuscripts and publish results, these local collaborators are left out under the assumption that they did not contribute “intellectually” to the project. We note that in some cases their names are mentioned in the acknowledgments section. However, their knowledge is instrumental for the development and successful completion of projects and should be accounted for as intellectual merit. Invite them to coauthor manuscripts; coauthorship can be especially relevant and career transforming for undergraduate, graduate students, and early-career researchers.

### Rule 2: Actively reach out to initiate Global South collaborations

Finding international collaborators can sometimes feel daunting, but chances are that someone else on the planet is doing similar work. When working in the Global South, many of the local researchers have been studying local ecosystems for years. Developing meaningful collaborations with these local researchers results in more efficient science because time and resources are saved; there is no need to replicate what has already been done. Identifying and taking advantage of “culture brokering” systems that are in place can facilitate efficacy of communication and bridging groups of people from differing cultural backgrounds [17]. For example, the Smithsonian Institution invests in fantastic infrastructure in Panama as proof of its long-term commitment to carry out biodiversity studies of the Isthmus and to support both local and international researchers. As mentioned previously, language can be a barrier, but English translators can be found almost everywhere. However, to rely on Global South partners to speak English is yet another way of imposing colonialism; researchers from the Global North should make an effort to learn the local language. Finally, if reaching out for collaborators in universities, most affiliates—professors, researchers, and graduate students—will have at least basic English knowledge. In other words, communication, although potentially difficult, is not impossible.

### Rule 3: Establish collaborations that are synergistic

Collaborations should be mutually beneficial. Good and strong collaborations take time to develop especially when considering language, administrative, distance, and time-constraint barriers. But, as mentioned before, research will likely be more impactful when it is collaborative and when cocreation of research ideas is set as a priority (see Rule 1). Often, Global South collaborators do not receive a fair share of the benefits derived from having their resources used by Global North scientists. The Bonn Guidelines [18] provide governments and stakeholders “with a transparent framework to facilitate access to genetic resources and ensure fair and equitable sharing of benefits.” Although not legally binding, researchers should make sure that they are aware and abide by local Bonn Guidelines. Here, local collaborators can help navigate administrative and political affairs that would otherwise be very difficult, if not impossible, for “outside” researchers. In addition, the Bonn Guidelines present a list of monetary and nonmonetary benefits—including research funding, milestone payments, sharing of results, contribution in education and training, participation in product development, contributions to the local economy, capacity building, technology transfer, and social recognition. Moreover, truly synergistic collaborations—incorporating, say, training of local partners—have less of a chance to be disrupted by global events. A fitting example, the Coronavirus Disease 2019 (COVID-19) pandemic has left many Global North scientists facing serious challenges and restrictions [19].

Synergy and benefit sharing should always be a priority. Researchers from the Global North must find a way to give back to the Global South based on their needs. For example, for a local collaborator who is an academic, coauthorship should always be granted. For collaborators who do not hold academic positions, and to whom publications are meaningless, researchers should still find meaningful ways to give back to them and their communities. Furthermore, Global North researchers should always make their findings accessible to local communities, including local collaborators. Part of this includes learning the local language or hiring someone who speaks both English and the local language as a point of contact at the field site. There is a need for Global North scientists to invest more in translating their research for local collaborators and their communities to understand. Note that while not speaking English may be considered a barrier, not speaking the local language is an equally important barrier as well [20]. When reviewing the available literature, non-English literature is often easily excluded. Local evidence can better explain biodiversity patterns and suggest conservation actions and policies, but these local data tend to be published in local languages.

### Rule 4: Abide by local written and unwritten rules

Showing respect in general is a good way to not perpetuate colonialism, so first and foremost, be respectful. Show culturally appropriate behavior, engagement, and respect for the local land and people (unwritten rules) and abide to local law (written rules). Local collaborators can help in navigating these rules. Government and field sites’ administration differ from place to place, and local collaborators are key figures to obtain the necessary paperwork and permits to sample, as they know best how their administration and government work. Even if there are no laws that require permits for exporting and collecting resources, researchers should avoid at all cost helicopter research [21] and should, at the very least, aim to deposit specimens at local institutions. This is an obligation at a growing number of Global North institutions (for example, at the Muséum National d’Histoire Naturelle, Paris). As a result, researchers are unable to sample if they do not deposit specimens in local institutions. Environmental agencies may demand deposition of type materials at local collections in the Global South. This is also happening in Panama as a result of the implementation of the Nagoya Protocol [22]. Unwritten rules may be as important as written rules. Aside from showing what is culturally appropriate behavior and engagement, local researchers can help identify field sites that are safe and appropriate for the proposed work versus sites that are areas for worship or conflict zones, and, thus, not available.

### Rule 5: Recognize and embrace differences in working culture

Working cultures differ around the world, and it is important to recognize and embrace these differences. Many scientists are overwhelmed by administrative and service work as well as heavy teaching responsibilities, aside from their scientific work. Global South researchers in particular have limited availability for research compared to Global North researchers due to cuts in research funding (e.g., in Brazil [23] and Mexico [24]). It is important to recognize that different collaborators may need different timelines and to accommodate for that. While we acknowledge that some sort of adjustment is justified as part of the adaptation to the new environment, stress, anxiety, disrespect, and dysfunctional communication—negative adjustment—will only worsen the situation and contribute to diminished work performance and interpersonal relationships.

Developing cultural competence may be a good way to thrive in multicultural and diverse working groups. This involves adopting behaviors that accomplish goals while at the same time minimize negative adjustment outcomes and maximize positive ones [25]. This involves openness, care, and respect toward local partners and the local community as well as toward the local culture and history. To experience a “culture shock” may be inevitable, but this is alright and even normal when familiar symbols and conceptualizations are lost, and we are met with another set of realities. Different stages of culture shock are recognized by different scholars; Oberg described 4 stages: honeymoon (everything is new), hostility/crisis (facing the realities of local life), recovery (learning local practices and language), and adjustment (enjoying the differences) [26]. Culture shock is not to be regarded as a “taboo” [27] and should instead be discussed in the open, perhaps as part of institutional coursework or mentorship; experiencing it is a rich learning experience and will increase resilience and adaptability.

Cultural competence intersects not only with nationality but also with other identities. For example, indigenous researchers and/or community leaders may carry added burdens associated with being “the trusted one” within their community or who may be put in difficult situations where they face conflicting cultural and professional responsibilities. Establishing a culturally safe environment requires recognizing intersectionality associated with working cultures and cultural identities and protecting and prioritizing the researchers’ integrity above all [6].

### Rule 6: Instill non-colonial collaborative research practices early on

For decades, Western science has been based on the expertise of colonized people. Charles Darwin collected specimens and developed his ideas around adaptation through natural selection during the second voyage of the HMS Beagle, a warship that in the first place was commissioned to circumnavigate and survey the globe. Imperialists considered the West with its science and medicine superior compared to the colonies and even Darwin [28] wrote that “At some future period, not very distant as measured by centuries, the civilised races of man will almost certainly exterminate, and replace the savage races throughout the world.” In recent years, there have been an increasing number of calls to decolonize science. The best way to remove colonial practices in research in the long term is (i) by teaching students what colonial research looks like, why colonial research practices are wrong; and (ii) by instilling a culture of non-colonial collaborative science in research groups. If students are planning to carry out field work or sample in the Global South, advisors should encourage students to collaborate with local scientists, including other students. Professors set the tone in their labs in terms of research practices and lab culture; thus, it is critical that professors set the example and do not perpetuate nor support colonial research practices. Exchange programs for students from the Global South to the Global North and vice versa could be helpful for establishing local connections early on in their scientific career. Such programs would increase collaborations when pursuing research in graduate school and further down in their careers, since a network would be already established.

### Rule 7: Use local infrastructure

Use local infrastructure where available and keep samples, data, and results on site. This is useful if samples are temperature- or time sensitive, and especially important if samples were extracted from protected species or sites. This is overall good practice and a good way to implement the Nagoya Protocol [22] for the utilization of genetic resources; this should be implemented for nongenetic resources as well (e.g., rocks). It is also a good way to enforce the Cartagena Protocol on Biosafety [29] for the overall protection of biodiversity and human health. In addition, using local infrastructure means that no samples need to be exported, thus is a good way to avoid hurdles in customs when returning to your country.

### Rule 8: Incorporate a capacity building component

Crucial for a healthy “global science” community is that Global South individuals and institutions acquire, improve, and retain skills, knowledge, equipment, and resources. Capacity building is often a vital, or required component of grant applications, but more than that it should be a no-brainer to anyone with a collaborative mindset. Excellence goes hand in hand with a focus on capacity strengthening, as was found by a meta-analysis of 170 studies from 130 separately funded research projects in Latin America, the Caribbean, the Middle East, Africa, and Asia [30]. We like to think about capacity building as a 2-way process—if it were true that the Global South only benefited from knowledge in the Global North—we would not be any different from 19th century imperialists. Again, we advocate for exchange programs for students to obtain gains from each other. By incorporating capacity building, some of the rules here suggested, such as introducing student exchange programs and using local infrastructure, will be easier to follow through.

### Rule 9: Be ethical and fair about publications and authorship

The names of many Global South local collaborators end up in the acknowledgments sections of peer-reviewed publications, whereas others are not even acknowledged. Have clear authorship guidelines for manuscripts. Being a local collaborator and participating in field work does not automatically warrant authorship, but when a local collaborator applies for permits, arranges logistics, has knowledge about remote field sites or microhabitats needed for the planned research, or identifies, say, plant hosts for fungal pathogens, then this should be rewarded with authorship. Similarly, allow for Global South collaborators to take leadership positions within projects, especially if there is a specific interest on their side; this could look many different ways, for example, Global South collaborator leading a few smaller projects locally while working synergistically with Global North collaborators to achieve a “bigger picture” goal. Moreover, peer-reviewed journals should actively participate in efforts to end helicopter research by adjusting their authorship and sampling guidelines. *Global Health Action*—an open-access journal about global health focusing on public health and policy issues—is an excellent example. In its instructions for authors is explicitly stated that manuscripts may be rejected by the handling editor upon initial screening when “[t]he study uses primary data that were collected by local researcher(s) in low- or middle-income countries and are not publicly available, but does not include any local researcher(s) as co-authors.” Other journals may follow suit to put an end to helicopter research.

### Rule 10: Make your research available through local dissemination

Making research results available to the local community is one of the best ways to give back and show gratitude [31]. Ways to do this are to publish in a local journal, perhaps in the local language, to write a science communication article for the community at large, to get in touch with the local press (local collaborators may help with this), or to create a brief YouTube video. In addition, it is a good idea to publish collaborative findings in open-access journals so that they are available to everyone, especially those in the Global South, who, in many cases, do not have access to papers behind an expensive paywall. Make sure to budget for open-access publications and broader impact activities in grant proposals.

## Conclusions

Helicopter research is an unfair and colonizing practice. As we move forward making science truly “global” and more socially just, these types of practices should disappear. A good way to start is at the individual and laboratory level; double-check institutional fieldwork practices, and make sure that projects are run fairly and inclusively. At the system level, journals should not allow helicopter research–practicing submissions, as recently suggested in *Geoderma*—a journal of soil science [21]. In addition, they could be more unequivocal on the definition of what grants coauthorship [32] such that intellectual contributions should also include local knowledge (e.g., field sites, microhabitats, language, etc.). Sampling permit numbers should be a requirement for publication, as are data availability statements, grant funding information, and sequence accession numbers. Finally, things are more likely to change faster if imposed by editors as a requirement for publication and by grant agencies as requirement for funding (including broader impacts that will benefit local communities and ensuring proposed activities are completed to secure future funding). Across all levels, true agency must be given to Global South collaborators; “charity” initiatives and “savior complex” behavior should be avoided at all costs. Helicopter research has been perpetuated for too long, and we now have the knowledge, the tools, and the social awareness to stop it.

The San people, with a hunter-gatherer history going back more than 20,000 years, now have a Code of Research Ethics [33] to assist research designing projects. These people have regularly been used to generate genomic-scale data, but, too many times, they were spoken down to or disrespected for their cultural sensitivities. Requirements are respect for the San culture, for people’s privacy, and for social practices; honest sharing of information in clear language; justice and fairness in terms of mostly nonmonetary benefits (see [18]); and care for the local needs. Finally, the Code focuses on the collective development of research ideas, by both researchers and the San. This is an approach that was developed by and for the San people. Beyond basic ethical considerations, each community will have a unique perspective and recommendations that may be different compared to the San Code of Research Ethics; researchers should be aware of and comply with the specific recommendations of each community [6]. Dr. Anne Pringle, President of the Mycological Society of America, reminded us that fieldwork in the Global South “isn’t development work, it is working together” (*Mycology from the Cloud* virtual meeting, 22 July 2020).
